# Mental Illness: A Deviation from Phenomenological, Rather than Moral, Norms?

**DOI:** 10.1093/jmp/jhaf032

**Published:** 2026-03-10

**Authors:** Adrian Downey

**Affiliations:** Queen’s University Belfast, Belfast, Ireland

**Keywords:** anti-psychiatry, consciousness, flow, mental illness, phenomenological psychopathology, well-being

## Abstract

Anti-psychiatrists contend that psychiatric practice is fundamentally misguided: it inappropriately medicalises difference by equating it with illness, resultantly forcing unwarranted “treatment” on the “mentally ill.” I explain why the most popular realist accounts of mental illness—naturalism, constructivism, and hybridism—are typically considered vulnerable to this moral problem. Then, I introduce “the phenomenological account” that promises to avoid it. The phenomenological account equates mental health with the being-body experiential mode, which is conducive to the flow experiences empirically demonstrated as constitutively necessary for subjective well-being. Mental illness, meanwhile, is equated with entrapment in the having-a-body mode. This precludes flow and, as such, subjective well-being. I explain why the phenomenological account is better placed to resolve the moral problem than its peers, thus providing a proof of principle demonstration of its potential to rebut the anti-psychiatry argument across the board. Finally, I defend the phenomenological account from three common objections.

## I. INTRODUCTION

One of the foundational ideals of the institution of medicine is that illness exists and ought to be treated. Whilst few would dispute the coherence of the concepts “health” and “illness” in the context of the (physical) body, there has always been heated debate over the legitimacy of such concepts when applied to matters mental. Accordingly, psychiatry—the branch of medicine dedicated to treating mental illness—is unique among medical specialities in consistently having its very *raison d’etre* subject to attack (Nasrallah, 2011; Burns, 2014). In this paper, by drawing on a body of work emphasising the personal level of experience, I suggest a novel means of shielding psychiatry from such attack.

I begin by outlining the *anti-psychiatry argument* impugning psychiatry’s legitimacy. It says that there is no such thing as mental illness and, thus, that psychiatry is best understood as an institution of social control; one that enforces the dictates of experience and behaviour deemed acceptable by polite society. Since psychiatry in its present form serves no legitimate medical purpose, it ought to be abolished (Section II). Next, I outline the three most prominent types of realist account of (mental) health/illness—naturalism, constructivism, and hybridism. And I explain why each is considered to come up short, in its defence of realism about mental health from the anti-psychiatry argument (Section III). A curious feature of these accounts of mental illness is that none focuses their attention on the personal level of experience; they each solely emphasise sub-personal biological goings-on and/or socio-cultural factors. I suggest a change of argumentative tack, maintaining that an emphasis on the experiential aspects of mental illness leads to an account well placed to rebut the anti-psychiatry argument. Hence, revealing this *phenomenological account* to possess an important advantage over the traditional realist fare (Section IV). I finish by defending the phenomenological account from three common objections (Section V) before wrapping things up in the conclusion (Section VI).

## II. THE ANTI-PSYCHIATRY ARGUMENT

The institution of psychiatry encompasses quite a few professional occupations: psychiatrist, clinical psychologist, mental health nurse, psychotherapist, social worker, and so on. Many of the activities of these various psychiatric professionals are organised around the idea that mental illness exists and ought to be alleviated. Consequently, patients coming under psychiatric remit are often assessed to determine whether they are mentally ill; if diagnosed as such, then they are given a variety of treatments aimed at helping them recover. This *prima facie* benign practice has come under sustained criticism from a variety of academics, (psychiatric) professionals, and activists. The specifics of this criticism can vary: some advert to psychiatry’s history of malpractice to impugn it, others to its authority to treat people against their will, yet others to its cosy ties with Big Pharma, and so on. But virtually all such arguments against psychiatry’s legitimacy question one of its most central contentions—that mental illness exists.

According to this *anti-psychiatry argument*,^1^ psychiatric diagnoses, while appearing scientifically medical in nature, constitute mere moral judgements reflecting dominant societal trends (Foucault, 1961/2001; Pickard, 2009; Szasz, 2011; Davies, 2013; Middleton and Moncrieff, 2019). Psychiatry, it is said, considers activities and experiences that adhere to societal norms *good* and so labels them “mentally healthy.” Those that diverge from the norm, meanwhile, are considered *bad* and so labelled “mentally ill.” Treatment is then engaged with the aim of reforming those exhibiting mentally ill symptoms and is considered successful when their activities and experiences are altered, such that they more closely resemble societal norms. This practice, critics maintain, conflates *difference* with *illness*, and thereby inappropriately medicalises harmless divergences from societal norms.

Consider in this vein the similarities between being ginger and being schizophrenic. Although people with ginger hair constitute an extremely small proportion of the world’s population, we do not consider them unhealthy for possessing an uncommon hair colour. This is because there is nothing inherently unhealthy about being different from other people. *Mutatis mutandis*, the anti-psychiatry argument goes, for people who exhibit the uncommon characteristics of schizophrenia. Granted, people rarely engage in conversation with hallucinated angels and daemons, say. But this does not mean that there is anything wrong with doing so, because being different does not equate to being ill. Accordingly, schizophrenia’s uncommon “way of being” is no more unhealthy than having ginger hair—its way of being is not symptomatic of an illness. Since there looks to be no medical basis for labelling uncommon ways of being “mentally ill,” psychiatry lacks a *raison d’etre*. Mental illness does not exist and neither, therefore, should psychiatry (or at least, not in its present form).

Many people object to the understanding of psychiatry just sketched. They argue that mental illness does exist, and that it causes harm to those suffering from it. Medicine, it is plausible to assume, ought to alleviate human suffering wherever possible. Thus, psychiatry is a legitimate medical institution—its existence is warranted because it serves to (help) alleviate the harm induced by mental illnesses. To make good on this conviction, however, psychiatry’s defenders must provide a medically sound account of mental health/illness. That is, they must provide principled reasons for thinking that (certain) uncommon ways of being constitute mental *illnesses* as opposed to mental *differences*. If the realist about mental illness cannot do this, then the anti-psychiatry argument succeeds—diagnoses of “mental illness” will have been revealed as disguised moral judgements after all. One way to assess realist accounts in this regard is by submitting them to the *moral problem* test, as I now explain.

### The Moral Problem

A key *desideratum* for realist accounts is that they categorise certain *prima facie* mentally ill ways of being as mental illnesses. There exist some uncommon ways of being which, if mental illness exists at all, will count as such.^2^ Schizophrenia would seem to constitute one such exemplar mental illness. It exhibits a number of invariant characteristics: schizophrenia has a strong genetic component (e.g., Trubetskoy et al., 2022), its presence is correlated with specific environmental circumstances (e.g., Stilo and Murray, 2019), its course tends to follow stable trajectories (e.g., Levine et al., 2011), and it responds in a predictable manner to pharmaceutical treatment (e.g., Barnes et al., 2020). Such invariant patterns are characteristic of medical illness. They are exactly what we would expect to find if schizophrenia was, indeed, a mental illness.^3^

Schizophrenia is characterised by *positive symptoms* like delusion and hallucination, *negative symptoms* like flattened affect and social withdrawal, and *disorganisation symptoms* like problems with speaking and understanding language (APA, 2023; WHO, 2023). This makes it look especially relevant to the anti-psychiatry argument, because schizophrenia’s symptoms are explicitly defined in terms of divergences from the norm: positive symptoms are labelled as such because they constitute additions to normal ways of being, and negative symptoms are labelled so because constituting privations from them.

Another important *desideratum* for realist accounts is that they distinguish between mentally ill and mentally well deviations from social norms. We earlier saw that, while both ways of being are equally uncommon, having schizophrenia and having ginger hair ought to be distinguished. The former looks to be an illness, whereas the latter does not. Or, to use a more realistic example, realist accounts ought to distinguish schizophrenia from homosexuality.^4^ Both are uncommon ways of being that diverge from societal norms, but only the former should be considered a mental illness.

These two *desiderata* for realist accounts and the examples used to illustrate them can be combined. Thus, we arrive at what I label “the moral problem”:

Can a given realist account of mental illness (a) successfully identify schizophrenic ways of being as mentally ill (b) without thereby identifying homosexual ones as such?

If a realist account fails to meet (a), then that would suggest it fails to track mental illness at all. Granted, such failure might not directly support the anti-psychiatry argument. But it would do so indirectly, by indicating that nothing corresponding to the term “mental illness” exists in nature. (As previously mentioned, if mental illnesses exist at all, then schizophrenia will most probably be one.)

A failure to fulfil (b) would also indicate that the realist account fails to track mental illness. Homosexuality is not a mental illness (as previously noted). If a realist account nevertheless deems homosexuality a mental illness, for the very same reasons that it deems schizophrenia one, then it follows that neither is a mental illness. A realist account’s failure at this juncture would directly support the anti-psychiatry argument; its diagnosis of schizophrenia as a mental illness would be shown just as baseless as its diagnosis of homosexuality as one.

The moral problem conveys the core of the anti-psychiatry argument while providing concrete examples with which to test realist accounts. Its successful resolution would constitute a proof of principle that one has alighted on a robust means of distinguishing mental health from illness. Consequently, an account capable of resolving the moral problem would look to be capable of successfully rebutting the more general anti-psychiatry argument. If the moral problem is not resolved, however, then the anti-psychiatry argument would be vindicated—an inability to resolve the moral problem would reveal psychiatry to have a moral problem. In the sections to follow, I first argue that extant realist accounts are vulnerable to the moral problem and so falter in the face of the anti-psychiatry argument (Section III). Then, I introduce a novel realist account of mental illness—the phenomenological account—which I argue can resolve the moral problem and, as such, promises to successfully rebut the anti-psychiatry argument (Section IV).

## III. THE STANDARD REALIST ACCOUNTS SUCCUMB TO THE MORAL PROBLEM

Realist accounts of mental health/illness in the analytic philosophy literature fit into three-fold categories: naturalism, constructivism, and hybridism. Naturalists locate the norms of mental health/illness in sub-personal biological (dys)functioning, constructivists in socio-cultural practises, and hybridists in an admixture thereof. In this section, I show why each such account fails the moral problem test, thus demonstrating their vulnerability to the anti-psychiatry argument.

Before continuing, it is important to highlight that the criticisms I raise in this section are very much not unique to the present treatment. Indeed, my pressing of the moral problem serves only to bring out familiar points in a manner bespoke to this paper’s argument. For a more descriptively and bibliographically exhaustive explication of the pros and cons of the various positions canvassed herein, see Murphy (2021).

### Naturalism and the Moral Problem

Naturalists believe that mental health/illness can be empirically discerned. That is, we can discover states of health or illness in the exact same way that we can discover new planets or species. Things being so, our diagnoses of health/illness are considered objective: a correct diagnosis reflects the make-up of the mind-independent natural world and is, accordingly, untainted by human value judgements. All extant naturalist accounts locate mental health/illness’ empirically discoverable norms in sub-personal biological functioning. Thus, they look to the nature and make-up of the human body to unveil the norms of health.

Christopher Boorse’s *biostatistical theory* (1975; 2014) constitutes a representative exemplar naturalist account. It defines “health” in terms of correct biological functioning. (Parts of) biological organisms function correctly when they fulfil the roles accorded to them by natural selection (i.e., when they assist survival and reproduction). And we can discern these natural functions by adverting to species-typical norms. “Illness,” meanwhile, is defined conversely—it is present when there occur deviations from species-typical natural functioning that hinder survival and reproduction.

Boorse provides an objective, value-free account of “health/illness” because the norms in which it traffics are of a distinctively scientific kind. Since it equates normality with species-typicality, Boorse’s account deploys a purely statistical understanding of “normal”—the mean around which traits of biological populations tend to hover. This is something that falls directly out of probability distributions, no human value judgements required.^5^ The account’s norms of harm, meanwhile, are supplied by evolution. Anything that negatively affects an organism’s evolutionary fitness—its ability to survive and reproduce—is *ipso facto* considered harmful to it. Such norms are naturalistically acceptable because they are evolutionary in nature; again, human value judgements play no role in their determination.

The biostatistical theory says that illness is present when a deviation from species-typical natural functioning (a statistical norm) negatively affects its fitness (an evolutionary norm). Given that we can empirically “read off” these norms from nature, it is concluded that health and illness exist as mind-independent phenomena awaiting scientific discovery. Human value judgements, therefore, play no role in this naturalist account.

The biostatistical theory would diagnose schizophrenia to be a mental illness. People with schizophrenia exhibit mental characteristics that are not species-typical. And their cognitive architecture reflects this by diverging from the human norm. These divergences undoubtedly hamper survival and reproduction abilities—people with schizophrenia often face difficulties in functioning independently, in maintaining romantic and social relationships, and, unfortunately, have a reduced life expectancy. Accordingly, the biostatistical theory deems schizophrenia a mental illness because its dysfunctioning mental mechanisms negatively affect survival and reproduction.

This very same reasoning also requires labelling homosexuality a mental illness, however. The biostatistical theory says that the natural function of sexual activity is procreation. Since homosexual practices thwart this natural function, they involve a deviation from species-typical natural functioning. What is more, this deviation is harmful to fitness. For one, engaging in homosexual practices can interfere with one’s survival prospects—in heteronormative societies, such behaviour can result in discrimination, ostracisation, and even death. Furthermore, and for obvious reasons, homosexual activity also interferes with one’s ability to reproduce. Since homosexuality involves dysfunctioning biological mechanisms that adversely affect one’s prospects of survival and reproduction, the biostatistical theory considers it a mental illness (Boorse, 1975; cf Kingma, 2007).

Boorse’s biostatistical theory counts both schizophrenia and homosexuality as mental illnesses. It cannot distinguish between the two and therefore fails the moral problem challenge. This failure is representative of a general problem facing all extant naturalist accounts, which look incapable “of establishing a satisfactory, science-based, distinction between normal and abnormal human functioning” (Murphy, 2021).

### Constructivism and the Moral Problem

Constructivists hold the polar opposite view to naturalists, maintaining that our understandings of health/illness are rooted solely in socially constructed human norms. Rather than discovering health/illness in nature, they contend that we bring it into being by deciding something is (un)healthy. A representative constructivist account is József Kovács’ (1998) (see also Cooper, 2020, for a cutting-edge version of the view). He defines mental health as follows:

The healthier a…mental characteristic, process, reaction is, the more it makes it possible for the individual to adapt to reasonable social norms without pain and suffering, and the longer, and happier a life it will be able to ensure him in that society. (Kovács, 1998, 38)

Mental illness, on this account, would then be defined conversely:

The less healthy a mental characteristic, process, reaction is, the less it makes it possible for the individual to adapt to reasonable social norms without pain and suffering, and the shorter, and unhappier a life it will be able to ensure them in that society.

*Prima facie*, this sounds a perfectly sensible definition of “mental health/illness.” But, under the surface lurks a serious issue—how to determine what a “reasonable social norm” is. For instance, why does schizophrenia’s violation of social norms make it count as a mental illness whereas homosexuality’s does not?

It might be contended that schizophrenia counts as a mental illness because it violates the social norms that we deem most appropriate. Homosexuality, meanwhile, does not, because it fails to violate appropriate social norms. Alternatively, we might decide that neither count as mental illnesses because neither diverges from “reasonable social norms.” Or, indeed, we might count both as mental illnesses. The problem is that, irrespective of how we carve things up, our constructivist determinations appear to lack any principled underlying rationale. The very idea of a “reasonable social norm,” that is, seems predicated on nothing more than simple *stipulation*, on ungrounded assertions that certain ways of being are mentally (un)healthy. Since Kovács’ account cannot provide a principled distinction between schizophrenia and homosexuality, it fails the moral problem test.^6^

This quandary presents itself for all constructivist accounts. It does so because, in maintaining that diagnoses of mental health/illness are based solely on human value judgements, constructivists straightforwardly accept anti-psychiatry’s understanding of psychiatry. Consequently, they struggle to provide principled reasons for thinking that certain (uncommon) ways of being are mentally ill, whereas others are not. This leaves constructivism seemingly incapable of successfully responding to the anti-psychiatry argument.

### Hybridism and the Moral Problem

Hybrid accounts of health/illness aim at a rapprochement of naturalism and constructivism. Jerome Wakefield’s *harmful dysfunction* account (1992) is perhaps the best known of such theories. He argues that mental illness is present when: (a) there exists a biological dysfunction; (b) which causes harm in a given socio-cultural niche. By adverting to both natural (dys)function and a socio-cultural understanding of “harm,” this account marries naturalist and constructivist insights.

The harmful dysfunction account would classify schizophrenia in the western world as a mental illness. We earlier saw that schizophrenia involves dysfunctioning biological mechanisms. Moreover, since this dysfunctioning obstructs flourishing in western society, it causes harm in that socio-cultural niche. For the very same reasons, however, the harmful dysfunction account also labels homosexuality a mental illness: homosexuality involves a dysfunctioning of biological mechanisms that, even in the most liberal of western societies, results in harm (Boorse, 2021; Dussault, 2021). In labelling both schizophrenia and homosexuality mental illnesses, the harmful dysfunction account falls afoul of the moral problem.

Hybrid theories writ large face such issues. This is primarily because, in juxtaposing naturalism and constructivism, they inherit the problems dogging their component theories. From naturalism, hybrid theories inherit an inability to non-arbitrarily distinguish between healthy versus ill deviations from biostatistical norms. And, from constructivism, hybrid theories inherit an inability to provide principled reasons for thinking that certain judgements of health/illness are correct, whereas others are misguided. In short, hybrid accounts effectively double-down on the aspects of their constituent theories that make them incapable of resolving the moral problem.

In this section I have explained why all three of the most prominent types of realism about mental illness fail the moral problem test. None of them can make a principled distinction between schizophrenia and homosexuality and, as such, each looks vulnerable to the anti-psychiatry argument. These highlighted problems—which, to reiterate, are very well-known (Murphy, 2021)—have as of yet resisted conclusive resolution. I shall now introduce a novel realist account of mental illness, the phenomenological account, which I maintain passes the moral problem test.

## IV. A PHENOMENOLOGICAL UNDERSTANDING OF MENTAL ILLNESS

All the main realist accounts of mental illness in the analytic philosophy literature focus their attention on sub-personal biological functioning and/or socio-cultural group judgement. While this focus may be justified in the context of physical (bodily) illnesses,^7^ it is rather puzzling where mental illness is concerned. Mental illness is, after all, *mental*. And its concomitant experiences largely constitute the source of much of its (seeming) harm. A bout of psychosis, for instance, is arguably distressing to its subject because *experienced* as such; not because it is caused by biological dysfunction and/or occurs in a certain type of society. Consequently, it is strange that extant realist accounts pay little heed to the personal (experiential) level of explanation.

The *Phenomenological Psychopathology* research tradition is an exception to this trend. It has birthed insightful analyses of mental illness predicated on taking seriously and elucidating its experiential character (Jaspers, 1913/1997; Blankenburg, 2001; Sass and Parnas, 2003; Fuchs, 2010; Svenaeus, 2013; Stanghellini et al., 2018; de Haan, 2020; Ratcliffe, 2020). Nevertheless, this literature has not yet focused explicitly on the question “what makes something a mental *illness*?” By appraising a representative phenomenological psychopathology account in the light of this question, I build on it and thereby arrive at a novel means of resolving the moral problem. This success, I argue, constitutes a proof of principle that realist accounts emphasising the personal level are better placed to refute the anti-psychiatry argument than their realist peers. Thus, their further pursuit and development is motivated.

### Fuchs’ Phenomenological Account

In his *The Psychopathology of Hyperreflexivity*, Thomas Fuchs (2010) provides an account of mental illness representative of the phenomenological psychopathology tradition writ large.^8^ He accords two phenomenological concepts a central role in his account:

1. *Being-body*: the feeling of pre-reflectively living through one’s body to interact with the world.
2. *Having-a-body*: the adoption of a detached, theoretical stance towards one’s body, such that it becomes just another object in the empirical world.^9^

Fuchs takes the being-body to be characteristic of mental health. When all is going well, we rarely explicitly reflect on our performance of a given activity. Instead, the body functions like a “transparent medium”—we live *through* it to interact absorbedly with our environment. This mentally well environmental interaction is pre-reflective in nature because we simply allow our embodied capacities to express themselves; we act without much in the way of self-conscious volition.

Reflective consciousness kicks in, Fuchs contends, when: there have been breakdowns in pre-reflective activity; or we wish to learn new pre-reflective bodily skills. For instance, I might recognise that my piano playing goes awry because I keep misplacing my finger. Through practice guided by such reflection, I come to place unthinkingly my finger in the requisite correct position. At which point, my piano playing can once more take on a pre-reflective character (Heidegger, 1927/1962; cf Ryle, 1946; Dreyfus and Dreyfus, 1986/2014).

While reflective consciousness is valuable in such circumstances, it is always true that you can have too much of a good thing. Fuchs argues that mental illness occurs when reflection becomes excessive. He fleshes out this idea with the following two concepts:

1. *Hyper-Reflexivity:* the adoption of an extremely reflective perspective on one’s embodied activities, one disrupting of pre-reflective worldly engagement. This signals a move away from the mentally healthy being-body to the mentally ill having-a-body mode of existence.
2. *Pathological Explication:* mentally ill subjects tend to *explicate the implicit*. Rather than “just doing” an activity, they focus on discerning and implementing the precise actions that must be undertaken. This disrupts and/or disables the ability to engage in pre-reflective activity.

Fuchs explains that a negative reciprocal interaction between these two phenomena inexorably leads to a move from the being-body to the having-a-body mode. A hyper-reflective perspective leads to pathological explication, which reinforces the hyper-reflective perspective, which leads to further pathological explication, and so on and so forth. Eventually, subjects are left incapable of living through their body to engage pre-reflectively with the environment. At which point, their body is experienced as just another object in the empirical world; an “opaque medium” that obstructs pre-reflective interaction.

Fuchs applies these general ideas to make intelligible a number of mental ailments: insomnia, obsessive-compulsive disorder, hypochondria, body dysmorphic disorder, and schizophrenia. In each case, the ailment is delineated in terms of its precise manner of drift from the being-body (to the having-a-body) mode.^10^ But, irrespective of its precise nature, hyper-reflexivity and pathological explication are invariantly considered the root cause of this drift. Since, according to Fuchs, mental illnesses writ large are characterised by such negative spiralling, his lesson is a general one, to be applied widely (see especially 2010, 251–252).

### Mental Illness and Its (Lack of) Relation to Flow

Fuchs provides no argument to support his equating of mental health with the being-body mode. This leaves his position wide open to anti-psychiatry critique (that he inappropriately maligns certain uncommon ways of being). If pressed on this point, though, I suspect Fuchs would say something like the following:

To someone who is familiar with classic cases and other severe cases of autism, and knows of the suffering that is associated with autism, it (the anti-psychiatry position) seems perverse. (Frith, 2008, 38)

Psychiatric professionals like the just quoted Utah Frith often justify their practices by contending that they aim at alleviating harm. Fuchs is a psychiatrist, and he will have encountered extremely troubled people in the clinic. Consequently, he likely takes being ensconced in the having-a-body mode to be bad for people because he has observed its catastrophic consequences firsthand. What is more, people trapped in the having-a-body mode themselves often contend that it is an unhappy, unfulfilling way to live: their engagement in hyper-reflection and pathological explication tends to be driven by the aim of gaining relief from their symptoms, and they often arrive at psychiatric clinics precisely because they seek help in this regard. Thus, though Fuchs may not explicitly mention it, the idea that near permanent habitation of the having-a-body mode is bad for human beings does enjoy *prima facie* support—it is bad because it causes and promulgates experiential suffering and harm.

When it comes to the physical body, our practice of labelling conditions that cause harm “unhealthy” is considered uncontroversial. Broken legs, stomach ulcers, and many other conditions cause pain and interfere with people’s ability to get on with their day-to-day lives. That these physical conditions are bad for people leads us to label them “unhealthy”, and to charge medicine with treating them so that we can be brought back to physical health. Excessive habitation of the having-a-body mode is also bad for people. Similarly to broken legs and stomach ulcers, it causes pain and interferes with people’s ability to get on with their day-to-day lives. That we label this experiential mode of being “unhealthy,” and charge psychiatry with its treatment, is therefore entirely consistent with the accepted, non-controversial practises of medicine writ large.

The *prima-facie* support for this argument in favour of realism about mental illness is bolstered by a great deal of empirical literature on subjective well-being. The late psychologist Mihaly Csikszentmihalyi dedicated his career to discerning what engenders subjective well-being in human beings. His conclusion—supported by a vast body of cross-cultural^11^ empirical evidence (Csikszentmihalyi, 2011, 2014a, 2014b)—was that *flow experiences* lie at the heart of subjective well-being:^12^

Flow is a subjective state that people report when they are completely involved in something to the point of forgetting time, fatigue, and everything else but the activity itself. It is what we feel when we read a well-crafted novel or play a good game of squash, or take part in a stimulating conversation. The defining feature of flow is intense experiential involvement in moment-to-moment activity. Attention is fully invested in the task at hand, and the person functions at his or her fullest capacity. (Csikszentmihalyi, Abuhamdeh, and Nakamura, 2014, 230)

This definition of “flow” could be applied *mutatis mutandis* to describe the being-body mode of experience; flow experience just is pre-reflective absorbed coping (see (Section V) for further defence of this claim). Consequently, Csikszentmihalyi et al.’s work on flow empirically supports Fuchs’ account of mental illness.

Experiencing flow is, as Csikszentmihalyi often puts it, *intrinsically rewarding*. Moreover, it is constitutively necessary for subjective well-being. Since the being-body mode is synonymous with flow, it follows that we have an empirically principled reason for agreeing with Fuchs that it is mentally healthy. You need to inhabit the being-body mode to undergo the intrinsically rewarding experiences constitutively necessary for subjective well-being; *ergo*, inhabiting the being-body and experiencing flow is good for you, and thereby healthy.

Conversely, near permanent entrapment in the having-a-body mode precludes being-body experiences. It stands in the way of flow and so precludes one from undergoing the intrinsically rewarding experiences constitutively necessary for subjective well-being. Since this experiential situation is bad for human beings, it follows that it is mentally unhealthy. Which means that, in addition to its *prima facie* support, Fuchs’ contention that it is unhealthy to be ensconced in the having-a-body mode also enjoys strong empirical support.

In short, Csikszentmihalyi et al.’s extensive work on flow supplies empirical evidence vindicating Fuchs’ assertion that habitation of the being-body mode is mentally healthy, whereas entrapment in the having-a-body mode is mentally ill. This *phenomenological account* provides principled, empirically grounded reasons for thinking that certain ways of being are mentally (un)healthy. To demonstrate this, let us now submit the phenomenological account to the moral problem challenge.

### The Phenomenological Account and the Moral Problem

If the phenomenological account does successfully pinpoint empirically grounded, medically defensible norms of mental health, then it ought to pass the moral problem test. And indeed it does.

Fuchs (2010) explains schizophrenia to be initially characterised by a breakdown in tacit, common-sense knowledge. Patients lose the ability to interact absorbedly with their environments; to inhabit the being-body mode. In a bid to return to the being-body, they then engage in the twin processes of hyper-reflection and pathological explication. However, this serves only to further estrange patients from the being-body mode. As this cycle repeats itself, patients move further and further away from the being-body, eventually ending up almost completely ensconced in the having-a-body mode. At this point, the most severe and distressing symptoms of schizophrenia become apparent:

The dissolution of the intentional arcs of perception, thinking, and action is so far advanced that the remaining fragments of perception, thought, or movement take on a strange, object-like character and finally appear to be imposed on the patient from the outside…It is not hard to see how typical ego disorders such as thought insertions or verbal hallucinations can develop from such [having-a-body] forms of experience. (Fuchs, 2010, 251)

Schizophrenia is characterised by near permanent habitation of the having-a-body mode. Accordingly, the phenomenological account deigns it a mental illness.

The phenomenological account, however, does not deign homosexuality a mental illness. People who are homosexual have zero issues inhabiting the being-body mode. They are perfectly capable of experiencing flow and, indeed, can induce it via homosexual activity. Moreover, homosexuality, *qua* homosexuality, does not broach hyper-reflection or pathological explication—people who are homosexual do not *ipso facto* get dragged by reflective brooding into entrapment in the having-a-body mode. Since homosexuality does not preclude the flow experiences constitutively necessary for subjective well-being, the phenomenological account does not consider it a mental illness. Before proceeding any further, let us elaborate this point.^13^

It is certainly true that in many societies (including the West), homosexuality often correlates with entrapment in the having-a-body mode, because homosexual people are more likely than heterosexuals to possess mental ill-health (e.g., King et al., 2018). However, our best empirical evidence tells us that this is because homosexual people are more likely to face adverse circumstances of the kind often conducive to the development of mental illness. When such risk factors are controlled for, it turns out that homosexual people are no more likely than heterosexual people to be mentally ill; for example, they are no more likely to experience a major depressive episode (Scott, Lasiuk, and Norris, 2017). Thus, there is nothing about the homosexual way of being that, in and of itself, begets entrapment in the having-a-body mode.

Furthermore, although I am not aware of any experiments which have directly tested the ability of homosexual people to experience flow, neither am I aware of any evidence whatsoever that they cannot. Given the sheer volume of experiments conducted on flow and the attendant huge sample-size, it is highly unlikely that homosexual people cannot experience flow and researchers have simply failed to notice this. Indeed, if anything, all the (anecdotal) evidence that we possess suggests the converse—the fact that people experience flow when immersed in work, music, sport, sex, and so on does not seem to depend on whether they are heterosexual versus homosexual.

This contrasts homosexuality with schizophrenia. In schizophrenia, the person’s very way of being is characterised by entrapment in the having-a-body mode; it is inherent to schizophrenia’s nature that one is largely restricted to having-a-body experiences. This entrapment precludes habitation of the being-body and, as such, stands in the way of flow:

For flow to be maintained, one cannot reflect on the act of awareness itself. The moment awareness is split so as to perceive the activity from “outside,” the flow is interrupted. (Csikszentmihalyi, 2014b, 138)

The hyper-reflective perspective attendant to schizophrenia’s having-a-body mode does not allow for experiences of flow. Since it precludes the experiences constitutively necessary for subjective well-being, schizophrenia is bad for human beings.

Put shorter, the schizophrenic way of being, *in and of itself*, precludes habitation of the being-body mode and so experiences of flow. Core to schizophrenia is an entrapment in the having-a-body mode that is inimical to the undergoing of being-body, flow experiences. Conversely, there is nothing about the homosexual way of being which, *in and of itself*, precludes habitation of the being-body mode and so experiences of flow. Our best extant evidence points to this conclusion, and we have no empirical evidence to the contrary. The schizophrenic way of being is not conducive to flow, the homosexual way of being is, and that is why the phenomenological account deems the former mentally ill and the latter mentally healthy.

The phenomenological account provides a principled distinction between schizophrenia and homosexuality. It correctly diagnoses schizophrenia as a mental illness without thereby also diagnosing homosexuality as one. Hence, the phenomenological account successfully resolves the moral problem.

### The Phenomenological Account and the Anti-psychiatry Argument

I have just shown that the phenomenological account succeeds in resolving the moral problem. But that it gets the right result in the case of schizophrenia and homosexuality does not necessarily entail that it gets things right more generally, where mental health/illness is concerned. Put otherwise, it is an open question whether the phenomenological account’s success will scale-up; whether its resolution of the moral problem results in a more general refutation of the anti-psychiatry argument. Thus, what I have heretofore provided is a proof of principle: that the phenomenological account can resolve the moral problem demonstrates its *potential* to successfully diffuse the anti-psychiatry challenge writ large.

In science, it is common for theorists to focus their efforts on model organisms or model phenomena. For example, research on cognition might proceed by focusing on the neural activity of rats when they engage in a specific cognitive task, like that of navigating a T-maze. Scientists focus on these model phenomena because they believe that, being models of the *explanandum* under investigation, the resultant findings and explanations generalise. For instance, the similarities between rat and human brains are such that, by studying how rats successfully navigate their environments, we *ipso facto* learn how human navigation works (and, indeed, how mammalian navigation in general proceeds).

As presented in this paper, the moral problem has effectively constituted a model proxy for the anti-psychiatry argument. Schizophrenia is a model divergence from the norm that is a mental illness, homosexuality one that is not, and determining whether realist accounts can distinguish between these ways of being enables us to subject them to the anti-psychiatry challenge. We have seen that, whereas the standard realist fare fails to distinguish between schizophrenia and homosexuality, the phenomenological account successfully resolves the model problem that is the moral problem. It follows that we have strong—though not indefeasible—reason to think that the phenomenological account’s success will generalise, such that it shall successfully distinguish between mentally healthy and mentally ill ways of being writ large. This potential puts the phenomenological account at a marked advantage to other realist theories (which, I have argued, look incapable of defusing the anti-psychiatry challenge).

The phenomenological account can be made to speak to a great deal of mental illnesses. It is based on Fuchs’ (2005, 2010, 2014, 2015) phenomenological thesis, which has been applied to elucidate many mental illnesses: autism spectrum disorder, bipolar disorder, body dysmorphic disorder, depression, hypochondria, insomnia, obsessive-compulsive disorder, and schizophrenia. Fuchs’ theory, in turn, is representative of the phenomenological psychopathology tradition, which comprises a rich body of work providing detailed accounts of a vast range of mental illnesses (Jaspers, 1913/1997; Blankenburg, 2001; Sass and Parnas, 2003; de Haan et al., 2013; Svenaeus, 2013; Stanghellini et al., 2018; de Haan, 2020; Ratcliffe, 2020). Nevertheless, the phenomenological account still has a long way to go before we can say that it constitutes a complete, successful account of mental illness; consider that the most recent iteration of the DSM lists just under three hundred mental illnesses (APA, 2023)!

What is most important to note, and what the phenomenological account uniquely adds to proceedings, is that it presents a neutral methodology for discerning the presence/absence of mental illness. The phenomenological account says that we should focus on the question of whether the (uncommon) way of being under scrutiny stands in the way of flow: if it does, then it probably is a mental illness, and if it does not, then it probably is not. Since the ability to experience flow constitutes a principled, empirically grounded norm of mental health, the phenomenological account supplies a non-arbitrary metric for appraising (uncommon) ways of being. Thus, it provides a means of diagnosing mental illnesses that is not vulnerable to the anti-psychiatry argument. This suggests that, with further development, the phenomenological account will result in a general realist theory of mental illness possessing of sound empirical, medical, and moral foundations.

## V. THREE COMMON OBJECTIONS

The phenomenological account tends to broach the following three objections: (1) it is question-begging; (2) it cannot account for flow experiences (putatively) occurring during habitation of the having-a-body mode; and (3) it has the absurd consequence that obviously mentally ill ways of being are deemed mentally healthy, because (seemingly) characterised by experiences of flow. I respond to each in turn.

### Begging-the-Question

The first objection maintains that the paper’s flawed set-up entails its entire argument is question-begging. The problem, specifically, is that it is *assumed* that schizophrenia is, and homosexuality is not, a mental illness. Then, when the phenomenological account ‘correctly’ distinguishes between these phenomena, it is deemed vindicated. But what is *really* in question is the very legitimacy of this distinction to begin with. Whether schizophrenia and homosexuality count as mental illnesses is something that varies widely across a range of spatiotemporal domains. Accordingly, we require reasons for thinking that this paper’s stance on schizophrenia and homosexuality is preferable to any other. Otherwise, it looks to reflect nothing more than a socio-culturally situated *value judgement.* In which case, the phenomenological account is revealed to be a disguised form of constructivism and, therefore, vulnerable to the moral problem after all.

I submit that the phenomenological account’s empirical evidence base obviates this question-begging charge. Granted, the initial conjecture that schizophrenia is a mental illness whereas homosexuality is not may lack independent support.^14^ Though Fuchs’ account may somewhat improve matters, by pinpointing a key difference between schizophrenic and homosexual modes of being, it still does not tell us why one mode of being would be mentally ill whereas the other is not. However, when the empirical work on flow is added to the mix, we do (finally) arrive at a vindication of the conjecture. This work points to its being the case that inhabiting the being-body mode is—invariantly, across all human cultures^11^ —constitutively necessary for subjective well-being. That empirical conclusion fully supports Fuchs’ thesis: habitation of the being-body mode is mentally healthy because it enables the goods of flow, and entrapment in the having-a-body mode is mentally ill because it precludes flow’s goods and so is bad for those subject to it. Hence, what initially began as a theoretical conjecture enjoying only intuitive, observational support ends up being an empirically well-confirmed hypothesis.

It follows that my argument is not question-begging. The phenomenological account provides a principled basis for labelling certain experiential ways of being “healthy” or “ill,” because its norms of mental health/illness are predicated on empirical work unearthing of invariant characteristics of human consciousness. Consequently, it does not amount to constructivism in disguise.^15^ To dispute this conclusion, one must grapple and find fault with the phenomenological account’s empirical evidence base.

### Flow and the Having-a-Body Mode

A second common objection points to instances of flow (putatively) undergone while subjects inhabit the having-a-body mode of experience. Models, bodybuilders, and yogis, for instance, all look to cultivate flow experiences by appraising their bodies as mere objects in the empirical world. Hence, the phenomenological account looks straightforwardly falsified.

Pre-theoretically, I agree that this looks like a damning objection. However, as is often the case in science, the empirical reality (of flow) belies appearances. It is simply not possible to experience flow while ensconced in the having-a-body mode:

Perhaps the clearest sign of flow is the experience of merging action and awareness. A person in flow does not operate with a dualistic perspective: one is very aware of one’s actions, but not of the awareness itself…(F)or flow to be maintained, one cannot reflect on the act of awareness itself. The moment awareness is split so as to perceive the activity from “outside,” the flow is interrupted. (Csikszentmihalyi, 2014b, 138)

On Csikszentmihalyi’s empirically arrived-at definition of “flow,” people who experience flow *ipso facto* inhabit the being-body mode of experience (cf Section III). In modelling, bodybuilding, and yoga, then, the only role the having-a-body mode *might* be playing is that of helping to inculcate being-body flow experiences. But, when people engaging in these activities actually experience flow, they are doing so because they inhabit the being-body mode. This objection to the phenomenological account therefore falters in the face of empirical evidence; to make good on it, its proponent must first identify relevant flaws in the empirical research on flow.

### Flow and Its Presence in Mental Illness^16^

A third common objection points to instances of being-body flow experience that look to constitute paradigmatic cases of mental *illness*, rather than health. Episodes of euphoric mania—prominently associated with bipolar disorder, but which can present in other conditions besides—are often adverted to in this vein. Those subject to euphoric mania can appear to inculcate flow by engaging in extremely self-destructive behaviour, often of a kind discordant with their values. For example, someone might destroy their marriage by engaging in homosexual extra-marital sex, even though they are in fact heterosexual and happily married. Clearly, experiencing episodes like this can be extremely harmful to a person; an assessment that, once the episode has passed, those subject to it will tend to agree with. Nevertheless, the phenomenological account looks to determine euphoric mania as an exemplar of mental health. Thus, a *reductio* ensues.

If the phenomenological account did deem euphoric mania to be mentally healthy (because constitutive of flow), then that would give us strong grounds for rejecting it. However, when experiences of flow and euphoric mania are submitted to cross comparison, any *prima facie* similarities between their experiential characteristics quickly evaporate.

Csikszentmihalyi (2014b) highlights six core attributes of flow:

1. There is a “merging of action and awareness” as you come to inhabit the being-body mode (cf (Section IV)).
2. Your attention is fully invested in the task at hand.
3. Ego-loss occurs, in the sense that you lose awareness of yourself as a self.
4. You feel completely in control of your situational surroundings.
5. The flow-inducing activity in which you are engaged affords concrete possibilities for action and feedback on their resultant consequences.
6. Your experience possesses an *autotelic* quality; it feels intrinsically rewarding, and the activities that induce it are engaged in for their own sake.

Episodes of mania, meanwhile, are characterised by the DSM-5 as follows:

A distinct period of abnormally and persistently elevated, expansive, or irritable mood and abnormally and persistently goal-directed behavior or energy, lasting at least 1 week and present most of the day, nearly every day (or any duration if hospitalization is necessary). (APA, 2023, 124)

Furthermore, to count as a manic episode, at least three—or four, if irritability is the sole characteristic mood change—from this list of seven symptoms must be exhibited:

1. An extremely inflated sense of ego.
2. Extremely minimal sleep requirements.
3. Bouts of excessive talking (or a felt urge to engage in them).
4. Racing thoughts and ideas.
5. Being easily distracted.
6. Psychomotor agitation (purposeless, repetitive actions typically driven by tension) or else sustained focus on a goal-directed task.
7. Engaging in very risky, often self-destructive activities.

Finally, it must also be the case that these changes in mood and symptoms have significantly adverse effects on the person’s well-being (of the kind perhaps meriting hospitalisation) or are accompanied by psychosis.^17^

Having outlined flow’s key characteristics and the symptomatology of an episode of (euphoric) mania, it should be crystal clear that the former has little to nothing in common with the latter. For one, and most pertinently, the symptoms of mania typically appear nowhere in the description of flow. Moreover, many of flow’s key characteristics in fact stand directly oppositional to those of mania: flow is characterised by ego-loss and a ‘merging of action and awareness’, whereas mania is often characterised by an enlarging of the ego and so an over-emphasis on one’s sense of self; flow is marked by attentive concentration upon the task at hand, mania often by distractibility; flow experiences are always intrinsically rewarding, while manic experiences can be marked by tension and irritability; flow enhances subjective well-being and does not occur concomitant with psychosis, whereas (euphoric) mania negatively affects subjective well-being and can be present during psychosis; and so on.^18^

In short, flow experiences do not amount to experiences of (euphoric) mania, nor are they present during (euphoric) manic episodes. Therefore, the phenomenological account does not absurdly deem episodes of euphoric mania paradigmatic instances of mental health. Which means that this objection supplies no reasons for thinking that the phenomenological account misguidedly diagnoses as mentally healthy clear-cut instances of mental illness.^19^

## VI. CONCLUSION

I began this paper by outlining the anti-psychiatry argument. It says that, when psychiatric practitioners diagnose people with a mental illness, they misguidedly equate difference with illness; certain uncommon ‘ways of being’ are inappropriately medicalised. Given that there is no medical basis for psychiatry, psychiatry in its present guise ought to be abolished. This challenge to psychiatry was operationalised in terms of the moral problem: if an account of mental illness cannot successfully demarcate schizophrenia and homosexuality, then it falls prey to the anti-psychiatry argument. Next, I explained that the three most prominent realist accounts of mental illness—naturalism, constructivism, and hybridism—all fail the moral problem test and so are vulnerable to the anti-psychiatry argument. I then introduced the phenomenological account, which equates mental illness with entrapment in the having-a-body mode of experience. The phenomenological account explains this entrapment to be bad, and so unhealthy, because it precludes the being-body flow experiences empirically demonstrated to be constitutively necessary for subjective well-being. Next, I showed that the phenomenological account passes the test posed by the moral problem, and thereby provided a proof of principle that, unlike other realist theories, the phenomenological account can successfully rebut the anti-psychiatry argument. Finally, I defended this account from three common objections. I therefore conclude that the phenomenological account provides a novel, empirically grounded realist understanding of mental illness that promises to put psychiatry (back) on a secure medical and moral footing.

## Footnotes

1 The argument to follow has been proffered under a variety of labels, including “anti-psychiatry,” “critical psychiatry,” “post-psychiatry,” “mad pride,” and “neurodiversity.” While there are differences between these various positions, because they all agree that psychiatry inappropriately medicalises uncommon “ways of being,” they all object to the term “mental illness.” For ease of exegesis, I use “anti-psychiatry” to refer to all such perspectives.

2 Setting things up this way brings with it a very real risk of question-begging, an issue I speak to later. If the reader is particularly bothered by this risk, I would suggest skipping straight to (Section V) before proceeding any further.

3 My thanks to an anonymous reviewer for bringing this point to my attention.

4 The case of homosexuality, unfortunately, constitutes a real-life proof of the anti-psychiatry argument. Until relatively recently, people exhibiting homosexual ways of being were labelled “mentally ill.” And they were forced to engage in barbaric “treatments” aimed at “reforming” their behaviour, such that it better cohered with heterosexual norms (Smith, Bartlett, and King, 2004; Dickinson, 2015a, 2015b). In this case, a perfectly healthy way of being was pathologised because it diverged from societal norms, specifically, heterosexual norms largely born of and justified by religious and, subsequently, psychiatric authority (Bullough, 1979/2020; Drescher, 2015).

5 See Kingma (2007) for effective pushback on this idea.

6 Note that Kovács (1998) himself freely admits this is a problem for his view.

7 See Carel (2018) for important dissent.

8 This is not to say that all phenomenological psychopathologists would agree with every single aspect of Fuchs’ account. But its general tenor is consonant with that of the accounts typically adumbrated within this tradition.

9 Fuchs references the work of Merleau-Ponty (1945/2013), Plessner (1975), and Polanyi (1967) as especially relevant to his use of these phenomenological concepts. But the concepts themselves ultimately originate in the work of Husserl (1952/1990), who is a foundational figure in phenomenology. (My thanks to an anonymous reviewer for helpful pointers in this regard).

10 For explanations of depression, bipolar disorder, and autism in the same vein, see Fuchs (2005, 2014, 2015).

11 This is an important aspect of Csikszentmihalyi and his collaborators’ work. The empirically supported thesis is that flow experiences are *invariantly* constitutively necessary for subjective well-being; this rings true for all human beings, irrespective of their particular physical and/or socio-cultural situatedness. Csikszentmihalyi et al. thus take themselves to have hit on a general empirical insight into the human condition.

12 Strictly speaking, Csikszentmihalyi et al. took themselves to show that flow is constitutively necessary for a happy, meaningful life. However, their arrival at this conclusion arguably constitutes over-reach because it is not obvious that subjective well-being equates to happiness or a meaningful life. A skilled torturer, for example, may cultivate flow-induced subjective well-being from their vocation, but that does not necessarily make their life meaningful. Although there exist defences of Csikszentmihalyi et al. from such objections (Van Zyl, Gaffaney, Van der Vaart, Dik, and Donaldson, 2024), I do not rely on them here. Instead, I simply adopt a weaker interpretation of Csikszentmihalyi et al.’s work—that it demonstrates flow is constitutively necessary for *subjective well-being*, rather than for a happy, meaningful life. (My thanks to an anonymous reviewer for raising this important point about Csikszentmihalyi et al.’s conclusion and encouraging me to amend my thesis in response to it).

13 My thanks to two anonymous reviewers for highlighting that my initial treatment of this point went by much too quickly.

14 Given that schizophrenia exhibits many of the hallmark characteristics of an illness (4–5), it is not clear that this claim does lack independent support. However, since: (a) proponents of the anti-psychiatry argument would dispute this idea; (b) the success of my argument does not hinge on it; I do not rely on the claim here.

15 Indeed, if anything, the phenomenological account probably fits best into the category of naturalism. It is predicated on empirical evidence—the qualitative reports of psychiatric patients and the qualitative-cum-quantitative work on flow. Given that this evidence is used to determine the norms of mental health/illness, the phenomenological account toes the naturalist line that these norms admit of empirical discovery. That said, I think it is best to conceptually distinguish naturalism from the phenomenological account for two reasons: (1) all extant naturalist accounts focus exclusively on sub-personal biological functioning, not personal level experience; (2) general mind-body problem considerations entail that a great deal of controversy uniquely surrounds the scientific-cum-metaphysical status of personal level experience. These stark differences suggest that conjoining naturalism and the phenomenological account would engender unnecessary confusion and/or a lack of clarity.

16 My thanks to an anonymous reviewer, whose pressing of this objection made me rethink the implications of my thesis.

17 If neither of these final criteria is met, and the episode lasts longer than four days but less than seven, then it is deemed an episode of hypomania.

18 It is worthwhile making a general comment on the role played by this “compare and contrast” strategy within the phenomenological account. As should by now be clear, the phenomenological account maintains that we determine the presence/absence of mental illness by determining whether a given way of being precludes flow. Accordingly, when employing the ‘compare and contrast’ strategy, we are not so much accepting/rejecting a given DSM definition as querying whether it concerns an inability to experience flow (only if it does *not* can we conclude that the DSM diagnosis is in error). The revisionary potential of the phenomenological account therefore lies not in questioning DSM definitions, but rather in questioning whether they point to flow’s absence. (My thanks to an anonymous reviewer for raising an interesting query in this regard.)

19 In response to the (euphoric) mania counterexample, I have here provided a negative argument to the effect that mania is not characterised by experiences of flow. For a positive reason to think that (euphoric) mania involves habitation of the having-a-body mode—and so is symptomatic of mental illness—see Fuchs (2014).

## References

[jhaf032-B1] American Psychiatric Association. 2023. Schizophrenia spectrum and other psychotic disorders. 2023. In Diagnostic and Statistical Manual of Mental Disorders. 5th ed., TR. Washington, DC: American Psychiatric Association.

[jhaf032-B2] Barnes T. R. , Drake R., Paton C., Cooper S. J., Deakin B., Ferrier I. N., Gregory C. J. et al, 2020. Evidence-based guidelines for the pharmacological treatment of schizophrenia: Updated recommendations from the British Association for Psychopharmacology. Journal of Psychopharmacology 34(1):3–78. 31829775 10.1177/0269881119889296

[jhaf032-B3] Blankenburg W. 2001. First steps toward a psychopathology of ‘common sense’. Philosophy, Psychiatry, and Psychology 8(4):303–15.

[jhaf032-B4] Boorse C. 1975. On the distinction between disease and illness. Philosophy and Public Affairs 5:49–68.

[jhaf032-B5] Boorse C. 2014. A second rebuttal on health. The Journal of Medicine and Philosophy: A Forum for Bioethics and Philosophy of Medicine 39(6):683–724. 10.1093/jmp/jhu035 25398760

[jhaf032-B6] Boorse C. 2021. Wakefield on Harm, Health, and Homosexuality (unpublished manuscript) [On-line]. Available: https://www.buffalo.edu/content/dam/www/romanell/2020-21/Boorse-Wakefield-Harm.pdf, 1–25 (accessed April 18, 2024).

[jhaf032-B7] Bullough V. 1979. Homosexuality, A History (From Ancient Greece to Gay Liberation). London, United Kingdom: Routledge.

[jhaf032-B8] Burns T. 2014. Our Necessary Shadow: The Nature and Meaning of Psychiatry. London, United Kingdom: Penguin.

[jhaf032-B9] Carel H. 2018. Phenomenology of Illness. Oxford, United Kingdom: Oxford University Press.

[jhaf032-B10] Cooper R. 2020. The concept of disorder revisited: Robustly value-laden despite change. Aristotelian Society Supplementary Volume, 94(1):141–61.

[jhaf032-B11] Csikszentmihalyi M. 2011. Flow: The Psychology of Optimal Experience. New York, NY: Harper Collins.

[jhaf032-B12] Csikszentmihalyi M. 2014a. Flow and the Foundations of Positive Psychology: The Collected Works of Mihaly Csikszentmihalyi. Dordrecht, Netherlands: Springer.

[jhaf032-B13] Csikszentmihalyi M. 2014b. Play and intrinsic rewards. In Flow and the Foundations of Positive Psychology: The Collected Works of Mihaly Csikszentmihalyi, ed. Csikszentmihalyi M., 135–53. Dordrecht, Netherlands: Springer.

[jhaf032-B14] Csikszentmihalyi M. , Abuhamdeh S., Nakamura J. 2014. Flow. In Flow and the Foundations of Positive Psychology: The Collected Works of Mihaly Csikszentmihalyi, ed. Csikszentmihalyi M., 227–38. Dordrecht, Netherlands: Springer.

[jhaf032-B15] Davies J. 2013. Cracked: Why Psychiatry Is Doing More Harm Than Good. London, United Kingdom: Icon Books.

[jhaf032-B16] de Haan S. 2020. Enactive Psychiatry. Cambridge, United Kingdom: Cambridge University Press.

[jhaf032-B17] de Haan S. , Rietveld E., Denys D. 2013. On the nature of obsessions and compulsions. Modern Trends in Pharmacopsychiatry 29:1–15. 25225014 10.1159/000351929

[jhaf032-B18] Dickinson T. 2015a. ‘Curing Queers’: Mental Nurses and Their Patients, 1935-74. Manchester, United Kingdom: Manchester University Press.

[jhaf032-B19] Dickinson T. 2015b. Out of DSM: Depathologizing homosexuality. Behavioural Sciences 5(4):565–75. 10.3390/bs5040565 PMC4695779 26690228

[jhaf032-B210] Drescher J. 2015. Queer diagnoses revisited: The past and future of homosexuality and gender diagnoses in DSM and ICD. International Review of Psychiatry 27(5):386–95. 26242413 10.3109/09540261.2015.1053847

[jhaf032-B20] Dreyfus H. , Dreyfus S. 2014. From Socrates to experts: The limits of calculative rationality. In Skillful Coping: Essays on the Phenomenology of Everyday Action and Perception, ed. Wrathall M. Oxford, United Kingdom: Oxford University Press.

[jhaf032-B21] Dussault A. C. 2021. The harmful-dysfunction account of disorder, individual versus social values, and the interpersonal variability of harm challenge. Medicine, Health Care and Philosophy 24(3):453–67. 33966154 10.1007/s11019-021-10021-8

[jhaf032-B22] Foucault M. 1961. Madness and Civilisation. London, United Kingdom: Routledge.

[jhaf032-B23] Frith U. 2008. Autism: A Very Short Introduction. Oxford, United Kingdom: Oxford University Press.

[jhaf032-B24] Fuchs T. 2005. Corporealized and disembodied minds: A phenomenological view of the body in melancholia and schizophrenia. Philosophy, Psychiatry and Psychology 12(2):95–107.

[jhaf032-B25] Fuchs T. 2010. The psychopathology of hyperreflexivity. The Journal of Speculative Philosophy 24(3):239–55.

[jhaf032-B26] Fuchs T. 2014. Psychopathology of depression and mania: Symptoms, phenomena, and syndromes. Journal of Psychopathology 20:404–13

[jhaf032-B27] Fuchs T. 2015. Pathologies of intersubjectivity in autism and schizophrenia. Journal of Consciousness Studies 22(1–2):191–214.

[jhaf032-B28] Heidegger M. 1927/1962. *Being and Time*. Oxford, United Kingdom: Blackwell.

[jhaf032-B29] Husserl E. 1952/1990. Ideas Pertaining to a Pure Phenomenology and to a Phenomenological Philosophy: Second Book Studies in the Phenomenology of Constitution. Dordrecht, Netherlands: Springer.

[jhaf032-B30] Jaspers K. 1913/1997. General Psychopathology. Baltimore, MD: Johns Hopkins University Press.

[jhaf032-B31] King M. , McKeown E., Warner J., Ramsay A., Johnson K., Cort C., Davidson O. 2018. Mental health and quality of life of gay men and lesbians in England and Wales. The British Journal of Psychiatry 183(6):552–58. 10.1192/bjp.183.6.552 14645028

[jhaf032-B32] Kingma E. 2007. What is it to be healthy? Analysis 67(294):128–33. 18270546 10.1093/analys/67.2.128 PMC2239248

[jhaf032-B33] Kovács J. 1998. The concept of health and disease. Medicine, Healthcare and Philosophy 1(1):31–9. 10.1023/a:1009981721055 11081280

[jhaf032-B35] Levine S. Z. , Lurie I., Kohn R., Levav I. 2011. Trajectories of the course of schizophrenia: From progressive deterioration to amelioration over three decades. Schizophrenia Research 126(1–3):184–91. 21093220 10.1016/j.schres.2010.10.026

[jhaf032-B36] Merleau-Ponty M. 1945/2013. Phenomenology of Perception. London, United Kingdom: Routledge.

[jhaf032-B37] Middleton H. , Moncrieff J. 2019. Critical psychiatry: A brief overview. BJPsych Advances 25(1):47–54.

[jhaf032-B38] Murphy D. 2021. Concepts of disease and health. In E. Zalta, *The Stanford Encyclopedia of Philosophy* [On-line]. Available: https://plato.stanford.edu/archives/spr2021/entries/health-disease (accessed April 18, 2024).

[jhaf032-B39] Nasrallah H. A. 2011. The antipsychiatry movement: Who and why. Current Psychiatry 10(12):4–53.

[jhaf032-B40] Pickard H. 2009. Mental illness is indeed a myth. In Psychiatry as Cognitive Neuroscience, eds. Broome M. Bortolotti L., 83–101. Oxford: Oxford University Press.

[jhaf032-B41] Plessner H. 1975. Die Stufen des Organischen und der Mensch. Berlin, Germany: De Gruyter.

[jhaf032-B42] Polanyi M. 1967. The Tacit Dimension . Garden City, NY: Anchor Books.

[jhaf032-B43a] Ratcliffe M. 2020. Existential feelings. In The Routledge Handbook of Phenomenology of Emotion, eds. T. Szanto and H. Landweer. London, United Kingdom: Routledge.

[jhaf032-B43] Ryle G. 1946. Knowing-how and knowing-that: The presidential address. Proceedings of the Aristotelian Society 46(1):1–16.

[jhaf032-B44] Sass L. A. , Parnas J. 2003. Schizophrenia, consciousness, and the self. Schizophrenia Bulletin 29(3):427–44. 14609238 10.1093/oxfordjournals.schbul.a007017

[jhaf032-B45] Scott R. L. , Lasiuk G, Norris C. M. 2017. Sexual Orientation and Depression in Canada. Canadian Journal of Public Health 107(6):e545–49. 28252373 10.17269/CJPH.107.5506 PMC6972326

[jhaf032-B46] Smith G. , Bartlett A., King M. 2004. Treatments of homosexuality in Britain since the 1950s—an oral history: The experience of patients. British Medical Journal 328(7437):427. 14751920 10.1136/bmj.37984.442419.EE PMC344257

[jhaf032-B47] Stanghellini G. , Broome M., Raballo A., Fernandez A. V., Fusar-Poli P., Rosfort R. (eds.) 2018. The Oxford Handbook of Phenomenological Psychopathology. Oxford, United Kingdom: Oxford University Press.

[jhaf032-B48] Stilo S. A. , Murray R. M. 2019. Non-genetic factors in schizophrenia. Current Psychiatry Reports 21(10): 100. 31522306 10.1007/s11920-019-1091-3 PMC6745031

[jhaf032-B49a] Svenaeus F. 2013. Anorexia nervosa and the body uncanny: A phenomenological approach. Philosophy, Psychiatry, & Psychology 20(1):81–91.

[jhaf032-B49] Szasz T. 2011. The myth of mental illness: 50 years later. The Psychiatrist 35(5):179–82.

[jhaf032-B50] Trubetskoy V. , Pardiñas A. F., Qi T., Panagiotaropoulou G., Awasthi S., Bigdeli T. B., Bryois J., et al 2022. Mapping genomic loci implicates genes and synaptic biology in schizophrenia. Nature 604(7906):502–8. 35396580 10.1038/s41586-022-04434-5 PMC9392466

[jhaf032-B51] van Zyl L. E. , Gaffaney J., van der Vaart L., Dik B. J., Donaldson S. I. (2024). The critiques and criticism of positive psychology: A systematic review. The Journal of Positive Psychology 19(2):206–35.

[jhaf032-B52] Wakefield J. 1992. The concept of mental disorder: On the boundary between biological facts and social values. American Psychologist 47(3):373–88. 1562108 10.1037//0003-066x.47.3.373

[jhaf032-B53] World Health Organisation. 2023. Schizophrenia or other primary psychotic disorders. In World Health Organisation, *International Statistical Classification of Diseases and Related Health Problems.* 11th ed. https://www.who.int/standards/classifications/classification-of-diseases (accessed April 18, 2024).

